# Energiekonflikte und Demokratiekrise. Eine radikaldemokratische Perspektive auf das Ringen um Gemeinwohlziele der Energiewende

**DOI:** 10.1007/s41358-021-00289-w

**Published:** 2021-09-13

**Authors:** Timmo Krüger

**Affiliations:** 1 Department of Regional planning, Brandenburgische Technische Universität Cottbus-Senftenberg, Konrad-Wachsmann-Allee 4, 03046 Cottbus, Deutschland; 2 Institut für Europäische Urbanistik, Bauhaus-Universität Weimar, Weimar, Deutschland

**Keywords:** Agonistischer Pluralismus, Deliberative Demokratie, Demokratiekrise, Energiewende, Gemeinwohl, Radikale Demokratie, Agonistic pluralism, Common good, Crisis of democracy, Deliberative democracy, Energy transition, Radical democracy

## Abstract

Der Klimawandel ist in öffentlichen Debatten (wieder) sehr präsent, spätestens seit den Protesten von *Fridays for Future*. Dies zeigte sich zuletzt in der Intensität der Kontroversen, in denen beispielsweise der Kohleausstieg auf gesamtgesellschaftlicher Ebene verhandelt wurde. Gleichzeitig nehmen lokale Konflikte um konkrete Energieprojekte zu. Die Virulenz der Energiekonflikte ist u. a. darauf zurückzuführen, dass sie oftmals Kristallisationspunkte für Auseinandersetzungen um die Anerkennung von bestimmten Interessen als legitime Gemeinwohlinteressen bilden. Diese Konflikte sind zunächst einmal eine Reaktion auf die Ausgestaltung der Energiewende und wirken auf diese zurück. Darüber hinaus stehen Energiekonflikte in einem wechselseitigen Wirkungsverhältnis mit der politischen Kultur. Sie sind Effekt und gleichzeitig Mitproduzent der in der Gesellschaft verhandelten Vorstellungen von Demokratie. Der vorliegende Artikel leistet einen zweifachen Beitrag zur Analyse der Gemeinwohlkonflikte in der Energiewende in Deutschland. Erstens werden empirische Ergebnisse der Energiewendeforschung im Hinblick auf die Auseinandersetzungen um die Definition und Priorisierung von Gemeinwohlzielen der Energiewende interpretiert. Ein zentraler Befund lautet, dass sich in den Energiekonflikten Phänomene einer Demokratiekrise zeigen, auf die in den Verhandlungen um konkrete Energieprojekte nicht adäquat reagiert werden kann. Zweitens werden die gewonnenen Erkenntnisse über Gemeinwohlkonflikte in der Energiewende demokratietheoretisch diskutiert. Dabei wird argumentiert, dass die fehlende Bereitschaft (von Akteur*innen der Regierungspolitik) zur Austragung von Konflikten um die Priorisierung von Gemeinwohlzielen der Energiewende ein zentrales Defizit darstellt. In Kombination mit der einseitigen Orientierung am deliberativen Demokratiemodell bei der Umsetzung von Energieprojekten verschärft dies die Tendenzen einer Demokratiekrise. Aus radikaldemokratischer Perspektive bedarf es agonistischer Streiträume, in denen politische Gegner*innen darum konkurrieren, ihre jeweiligen Energiewende-Visionen durchzusetzen.

## Abstract (en)

Climate change is very present in public debates (again), at the latest since the Fridays for Future protests. This manifested recently in the intensity of societal controversies in which, for example, the coal phase-out was negotiated. At the same time, local conflicts over specific energy projects are on the rise. Energy conflicts are particularly acute, among other reasons, because they often form focal points for disputes over the recognition of certain interests as legitimate common good interests. These conflicts are first and foremost a reaction to the design of the energy transition and, at the same time, have an impact on it. In addition, these conflicts are in a reciprocal relationship with the political culture of a society. They are both an effect and a co-producer of the ideas of democracy negotiated in society. This article makes a twofold contribution to the analysis of common good conflicts in the German energy transition. First, empirical results of energy transition research are interpreted with regard to the struggles over the definition and prioritization of common good goals of the energy transition. A key observation is that energy conflicts reveal phenomena of a crisis of democracy that cannot be adequately addressed in negotiations over concrete energy projects. Second, the findings on common good conflicts in the energy transition are discussed in the light of democratic theory. It is argued that the lack of willingness (on the part of government policy actors) to engage in conflicts over the prioritization of common good goals of the energy transition is a central deficit. In combination with a one-sided orientation towards the deliberative model of democracy (in the processes of implementing energy projects), this exacerbates the tendencies of a crisis of democracy. From a radical democratic perspective, there is a need for agonistic arenas of dispute in which political opponents compete to implement their respective energy transition visions.

Die Kontroversen um den Kohleausstieg, um den Mindestabstand für Windräder und um die Kaufprämie für Autos zeigen: Die Energiewende ist umkämpft.^1^ Mit der AfD (Alternative für Deutschland) ist mittlerweile in allen deutschen Parlamenten eine Partei vertreten, die jeglicher Klimaschutzpolitik ablehnend gegenübersteht. Auch im außerparlamentarischen Bereich wächst der Protest gegen die Energiewende, insbesondere in Form von Bürger*inneninitiativen gegen den Bau von Windkraftanlagen, die sich mit Hilfe des Dachverbandes *Vernunftkraft* professionalisieren und vernetzen. Gleichzeitig entwickelten sich in den letzten Jahren mit *Fridays for Future, Ende Gelände* und *Extinction Rebellion* neue Formen des Klimaprotestes mit großen Mobilisierungserfolgen und hoher Medienpräsenz. Zwischen diesen beiden Polen steht eine Bundesregierung, die sich zwar einerseits zum Klimaschutz bekennt, andererseits aber ein Kohleausstiegsgesetz verabschiedet, mit dem die Ziele des Pariser Klimaabkommens nicht eingehalten werden können (Oei et al. 2020, S. 3). Die Auseinandersetzungen um Windkraft und Kohleverstromung stehen aktuell im Zentrum der Aufmerksamkeit, aber auch in anderen Bereichen der Energiewende nimmt seit einigen Jahren die Häufigkeit und Intensität von Konflikten zu. Diese Konflikte sind zunächst einmal eine Reaktion auf die Ausgestaltung der Energiewende und wirken auf diese zurück, d. h. sie beeinflussen die Geschwindigkeit und den Charakter der Energiewende. Darüber hinaus stehen Energiekonflikte in einem wechselseitigen Wirkungsverhältnis mit der politischen Kultur einer Gesellschaft. Die Energiewende stellt eine besonders sichtbare und flächenintensive Antwort auf die Herausforderungen der Klimakrise dar. In diesem Sinne sind Energiekonflikte Effekt und gleichzeitig Mitproduzent der in der Gesellschaft verhandelten Vorstellungen von Demokratie. Ihre gegenwärtige demokratiepolitische Bedeutung zeigt sich darin, dass sie oftmals (gerade auf lokaler Ebene) Kristallisationspunkte für Auseinandersetzungen um die Anerkennung von bestimmten Interessen als legitime Gemeinwohlinteressen sind (Becker et al. 2016; Messinger-Zimmer und Zilles 2016).

Der Begriff des Gemeinwohls wird in der Wissenschaft sehr unterschiedlich verwendet. Der kleinste gemeinsame Nenner besteht laut Moss et al. (2009, S. 38) darin, das Gemeinwohl als Interesse aller beziehungsweise der Allgemeinheit zu definieren, im Gegensatz zu den Partikularinteressen von Einzelnen oder Gruppen. Was die Bestimmung des Gemeinwohls betrifft, hat in den liberalen, westlichen Gesellschaften ein Wandel von der substanziellen hin zur prozeduralen Festlegung des Gemeinwohls stattgefunden (Seubert 2011, S. 94). Die Vorstellung, es gäbe ein objektiv bestimmbares, ideales Gemeinwohl, das als Richtschnur und Vergleichsfolie für reale Politik fungieren könne, wird somit abgelöst durch die Annahme, das ideale Gemeinwohl sei nur als – permanent neu zu beurteilendes und stets revidierbares – Ergebnis eines demokratischen Willensbildungsprozesses denkbar. Diese Entwicklung hängt zunächst einmal mit einer empirisch beobachtbaren Pluralisierung von Vorstellungen des Guten zusammen (ebd.), aber auch mit dem normativen Urteil, die Offenheit der Gemeinwohldefinition als Eigenwert zu begreifen (Schuppert 2002, S. 22). Die Folge ist, dass eine verbindliche Bestimmung des Gemeinwohlbegriffs ausgeschlossen wird. Gemeinwohlziele können demnach nicht universell bestehen, sondern werden prozedural, d. h. in gesellschaftlichen Auseinandersetzungen, festgelegt (Moss et al. 2009, S. 41; Becker et al. 2016, S. 41). Daraus ergibt sich die permanente Aufgabe, konkrete Gemeinwohlziele zu benennen. In der Frage, wie diese Aufgabe adäquat bewältigt werden kann, konkurrieren in westlichen Gesellschaften (sowohl in der Wissenschaft als auch in der Politik selber) in den letzten Jahrzehnten vor allem Ansätze des Liberalismus und des Republikanismus. Der im zweiten Kapitel vorgestellte Vorschlag der deliberativen Demokratie knüpft sowohl an liberale als auch an republikanische Denktraditionen an (Habermas 1997, S. 285; Seubert 2011, S. 98). Ich selber schlage, ebenfalls im zweiten Kapitel, eine hegemonietheoretische Verwendung der Begriffe Partikularinteresse und Gemeinwohl vor. Dabei baue ich primär auf Laclau und Mouffe (2001) auf und deren diskurstheoretische Reformulierung des Hegemoniebegriffs von Gramsci (1971). Die hegemonietheoretische Annahme lautet, dass es keine neutrale oder vernunftbasierte Unterscheidung von Gemeinwohl- und Partikularinteressen geben kann (Fraser 1990, S. 70ff.). Schließlich basiert eine solche Unterscheidung stets auf einer spezifischen Weltanschauung, die sich im Konflikt gegen andere Weltanschauungen durchgesetzt hat und (kollektive) Identitäten formt, aus denen heraus eine allgemeine Geltung für bestimmte Positionen postuliert wird (Bond 2011, S. 165; Mouffe 2015, S. 100). Die Unterscheidung zwischen Partikularinteresse und Gemeinwohl verstehe ich somit als das Ergebnis von Kämpfen um Hegemonie. Aus dieser Perspektive lauten die zentralen Fragen, wie Interessen universalisiert werden, d. h. wie bestimmte Interessen als Allgemeininteressen der Gesellschaft postuliert und durchgesetzt werden und welche Partikularinteressen im dominanten Verständnis von Gemeinwohl privilegiert eingeschrieben und welche marginalisiert sind?

Der vorliegende Artikel leistet einen zweifachen Beitrag zur Reflexion dieser Gemeinwohlkonflikte. Erstens werden empirische Ergebnisse und Debatten der Energiewendeforschung im Hinblick auf die Auseinandersetzungen darum, welche Interessen als legitime Gemeinwohlinteressen anerkannt werden und welche nicht, interpretiert (Kapitel 1). Dabei wird argumentiert, dass sich in den Energiekonflikten Phänomene einer Demokratiekrise zeigen. Konkret manifestiert sich das in Tendenzen einer Legitimations- und Repräsentationskrise sowie in der Verknüpfung von Kritik an der (Umsetzung der) Energiewende mit der Ablehnung pluralistisch-demokratischer Institutionen. Vor diesem Hintergrund werden, zweitens, die gewonnenen Erkenntnisse über Gemeinwohlkonflikte in der Energiewende demokratietheoretisch diskutiert (Kapitel 2). Dabei wird die These begründet, dass die Tendenzen einer Demokratiekrise durch die Kombination aus fehlender Bereitschaft zur Austragung von Konflikten um die Priorisierung von Gemeinwohlzielen der Energiewende plus einseitiger Orientierung am deliberativen Demokratiemodell – bei gleichzeitig mangelhafter Umsetzung desselben – verstärkt werden. Aus radikaldemokratischer Perspektive läge der Schlüssel für eine konstruktive Bearbeitung von Gemeinwohlkonflikten darin, die Konfrontation zwischen konkurrierenden Energiewende-Visionen zu forcieren und in einer Weise zu organisieren, die mit einem pluralistischen Verständnis von Demokratie kompatibel ist.

## Bestandsaufnahme der Gemeinwohlkonflikte in der Energiewende

Ich beschränke mich auf die Energiewende in Deutschland – zum einen, weil die Menge an Forschung allein zu diesem räumlich eingeschränkten Untersuchungsfeld schon überbordend ist, zum anderen, weil sie als eine der Energiewenden mit globaler Relevanz angesehen wird. Auch wenn die Energiewende in anderen Ländern teilweise weiter fortgeschritten ist, gilt die Energiewende in Deutschland für viele Akteur*innen als Experiment, wie ambitioniert, in welchem Zeitraum und mit welchen Konsequenzen eine Energiewende in einem bevölkerungsreichen Industrieland umgesetzt werden kann – und erhält dementsprechend auch vergleichsweise viel internationale Aufmerksamkeit (David und Gross 2019; Quitzow et al. 2016, S. 165 f.). Die Energiewende in Deutschland – so die gängige Definition – umfasst den Wandel von einer vornehmlich auf fossilen Energieträgern sowie Kernenergie basierenden Energieversorgung zu erneuerbaren Energien und höherer Energieeffizienz (Eichenauer 2018, S. 315; Quitzow et al. 2016, S. 163 f.). Bis dato ist die Energiewende allerdings primär eine Stromwende. In den Sektoren Verkehr und Wärme haben bislang weit weniger Transitionsprozesse und Emissionsreduktionen stattgefunden (Fraunhofer IWES/IBP 2017; Umweltbundesamt 2019, S. 71). Als Konsequenz daraus konzentriert sich auch die bisherige Energiewende-Forschung größtenteils auf den Strombereich.

Die Energiewende in Deutschland wird zwar oftmals als gesellschaftlicher Konsens bezeichnet, da die Zustimmungswerte – bei der Frage nach der Beurteilung der Energiewende im Allgemeinen – in der Bevölkerung konstant hoch sind (BMU et al. 2019, S. 28 ff.). Gleichzeitig nimmt die Intensität und Häufigkeit von Konflikten um konkrete Energieinfrastrukturprojekte zu (Eichenauer 2018, S. 316; Reusswig et al. 2016, S. 225). Dies führt zu der Frage, ob man tatsächlich einen breiten Konsens unterstellen kann, auf welche (mit der Energiewende verknüpften) Gemeinwohlziele sich dieser genau bezieht und inwieweit eine Einigkeit über Fragen der Ausgestaltung der Energiewende besteht (Radtke 2020, S. 98). Relativ unstrittig – sowohl innerhalb des politischen Systems als auch in der breiten öffentlichen Debatte – ist der Ausstieg aus der Atomkraft. Ausgerechnet der Atomausstieg wird parteiübergreifend nicht mehr angetastet, nachdem die Auseinandersetzung um die nukleare Energieversorgung über Jahrzehnte hinweg den emblematischen Energiekonflikt bildete. Angesichts der Nuklearkatastrophe in Fukushima nahm die schwarz-gelbe Regierung im Jahr 2011 ihre erst im Vorjahr beschlossenen Laufzeitverlängerungen – mit denen sie die Vereinbarung zwischen der rot-grünen Vorgängerregierung und den Energieversorgungsunternehmen bzgl. Reststrommengen der Atomkraftwerke revidiert hatte – wieder zurück. Seitdem gibt es keine ernsthaften Versuche, den Atomausstieg erneut rückgängig zu machen. Diese Entscheidung wird bislang von nahezu allen relevanten Akteur*innen als endgültig akzeptiert (Joas et al. 2016, S. 48) – eine Ausnahme bildet die AfD. Nimmt man aber den über den Atomausstieg hinaus gehenden Umstieg auf eine vorwiegend auf erneuerbare Energieversorgung insgesamt in den Blick, rücken ganz andere Konflikt- und Akteurskonstellationen in den Blick und dementsprechend zeigt sich die Interessenlage weit unübersichtlicher. So spricht beispielsweise Chemnitz (2018) vom „Mythos des Energiewendekonsenses“. Ihrer Meinung nach hat es nie einen länder- und parteiübergreifenden Konsens gegeben, was auch die Koordinations- und Steuerungsprobleme bei der Umsetzung der Energiewende im Föderalismus erkläre (ebd., S. 157 f.).

Im Folgenden erörtere ich die Auseinandersetzungen um die Definition und Priorisierung von Gemeinwohlzielen der Energiewende (1.1). Dabei argumentiere ich, dass einerseits nicht explizit über die Gemeinwohlziele der Energiewende verhandelt wird, es andererseits aber harte Konflikte um de facto Ziel-Priorisierungen durch Politikinstrumente und um die Realisierung von konkreten Energieprojekten gibt. Anschließend gebe ich einen Überblick über die virulenten Konflikte um die Umsetzung der Energiewende (in denen auch Auseinandersetzungen um die Ziele ausgetragen werden) und arbeitete Tendenzen einer Demokratiekrise heraus. In diesen Konflikten werden konkurrierende Gerechtigkeitsansprüche artikuliert. In meiner Darstellung differenziere ich zwischen den Dimensionen Verteilungs- (1.2), Anerkennungs- (1.3) und Verfahrensgerechtigkeit (1.4). Die strukturierte Zusammenschau der Konflikte, Phänomene der Demokratiekrise und artikulierten Gerechtigkeitsansprüche bildet eine empirisch gesättigte Problemstellung, zu der mit den demokratietheoretischen Überlegungen im zweiten Kapitel eine These entwickelt wird.

### Vage Gemeinwohlziele und Konflikte um de facto Ziel-Priorisierungen

Von Seiten der Bundesregierung werden (über mehrere Wahlperioden hinweg, also auch von verschiedenen Regierungskoalitionen) regelmäßig Versorgungssicherheit, Bezahlbarkeit sowie Klima- und Umweltschutz als vermeintlich gleichberechtigte Ziele der Energiewende postuliert (Quitzow et al. 2016, S. 163; Umbach 2015, S. 6 ff.). Weitere Ziele gelten als nachgeordnet (z. B. Verringerung der Abhängigkeit von Energieimporten, Joas et al. 2016) oder werden selten artikuliert, obwohl sie teilweise stark handlungsleitend sind (z. B. Schutz bestimmter Industriezweige, allen voran der Automobilindustrie). Die von der Bundesregierung ausgegebenen Gemeinwohlziele wurden rechtlich verankert, z. B. im *Erneuerbare-Energien-Gesetz* (EEG) oder im *Energiewirtschaftsgesetz* (EnWG) (Becker et al. 2016, S. 42). In diesen Gesetzen wird das energiepolitische Zieldreieck, bestehend aus Versorgungssicherheit, Bezahlbarkeit sowie Klima- und Umweltschutz, durch weitere Ziele – wie z. B. die „Weiterentwicklung von Technologien zur Erzeugung von Strom aus erneuerbaren Energien“ (BMJV 2017; § 1) und die „Sicherstellung eines wirksamen und unverfälschten Wettbewerbs bei der Versorgung mit Elektrizität und Gas“ (BMJV 2005; § 1) – ergänzt. Die verschiedenen, sich zumindest auch potenziell widersprechenden, Ziele stehen unvermittelt nebeneinander, d. h. es erfolgt keine Priorisierung.

Vage Zielformulierungen und der Verzicht auf Priorisierung können den Vorteil haben, dass verschiedene Gruppen die Ziele unterschiedlich interpretieren können, was die Kompromissbildung vereinfachen kann (Joas et al. 2016, S. 50). So war der Photovoltaik-Boom in Deutschland vermutlich nur möglich, weil er ganz unterschiedliche Interessen bediente. So bekamen Landwirt*innen neue Einnahmemöglichkeiten, Ingenieur*innen ein neues Arbeitsfeld, Kommunalpolitiker*innen eine neue Strategie für Strukturpolitik und Umweltorganisationen eine klimafreundlichere Stromversorgung (ebd.). Insofern sind vage Ziele nicht per se problematisch, sondern können unter bestimmten Rahmenbedingungen durchaus eine sinnvolle Strategie darstellen. Allerdings birgt ein instrumenteller Umgang mit Zielen – d. h. vage Zielformulierungen und ein Verzicht auf Priorisierung als Mittel zur Konfliktvermeidung – stets die Gefahr, zu unwirksamen, kontraproduktiven oder teuren Maßnahmen zu führen (ebd., S. 50 f.), wie aktuell am *Kohleausstiegs*- und *Strukturstärkungsgesetz* beobachtet werden kann (siehe Kapitel 1.2).

Auch wenn es auf Bundesebene kaum Auseinandersetzungen um die Formulierung von Zielen der Energiewende gibt, so gibt es harte Konflikte um Politikinstrumente wie das EEG, in denen es zu de facto Ziel-Priorisierungen kommt. Die Konflikte verlaufen zwischen Parteien, Energiekonzernen, Stadtwerken, Interessensverbänden, Bundesländern, Bundesministerien und Umweltorganisationen. Als Ergebnis dieser Konflikte hat laut Umbach (2015, S. 28) eine Zweck-Mittel-Umkehrung stattgefunden, so dass der Ausbau der erneuerbaren Energien nicht mehr primär Mittel ist, sondern zum eigentlichen Ziel wird – und der Klimaschutz dagegen weniger Priorität genießt. Die geringere Priorisierung des Klimaschutzzieles zeigt sich u. a. darin, dass vor allem die Stromwende vorangetrieben wird, die Bereiche Mobilität und Wärme jedoch weitestgehend außen vor bleiben, obwohl z. B. mit der Sanierung von Gebäuden eine „wesentlich größere Hebelwirkung bezüglich der CO_2_-Reduzierung“ (Umbach 2015, S. 27) erzielt werden könnte (im Vergleich zur Subventionierung des Stroms aus erneuerbaren Energien). Aber auch innerhalb des Stromsektors wirkt sich die Zweck-Mittel-Umkehrung aus. So ist zwar der Zuwachs an erneuerbaren Energien enorm, jedoch ist die Kohleverstromung lange Zeit relativ konstant geblieben (David und Gross 2019). Dabei wäre ein schneller Kohleausstieg die entscheidende Bedingung für eine signifikante Verringerung der Treibhausgasemissionen (Biesecker und von Winterfeld 2016, S. 36). Über alle Sektoren hinweg zeigt sich die geringere Priorisierung des Klimaschutzzieles an der Vernachlässigung von Suffizienzstrategien^2^, obwohl diese zu einer vergleichsweise schnellen Reduktion von Emissionen und Ressourcenverbrauch führen könnten (Biesecker und von Winterfeld 2016, S. 35). Sie gelten als politisch nicht durchsetzbar, weil sie mit dem Primat des Wirtschaftswachstums kollidieren. Dagegen gilt die Förderung von erneuerbaren Energien und Elektroautos als Möglichkeit, sogenanntes grünes Wachstum zu generieren.

Insgesamt zeigt sich, dass das Fehlen einer expliziten Ziel-Priorisierung zu Zielverfehlungen führt, beispielsweise in Bezug auf die Senkung von Treibhausgasemissionen (Chemnitz 2018; Umbach 2015, S. 16 f.). Das Klimaziel für 2020 hat Deutschland allein auf Grund des Wirtschaftseinbruchs durch die COVID-19-Pandemie erreicht und es ist davon auszugehen, dass mit der Erholung der Wirtschaft auch die Emissionen rasch wieder steigen werden. Die fehlende Ziel-Priorisierung hat weiterhin zur Folge, dass in den Auseinandersetzungen um die Umsetzung der Energiewende die Konflikte um ihre Ziele ausgetragen werden (Chemnitz 2018), worauf ich in den folgenden Unterkapiteln 1.2 bis 1.4 eingehe.

### Konflikte um Verteilungsgerechtigkeit

Die klassische Definition von Verteilungsgerechtigkeit bezieht sich auf das Ziel einer gerechten Verteilung von Ressourcen und Wohlstand (Fraser 2003, S. 7). Im Kontext von Klimakrise und Energiewende sind drei Ergänzungen aus der Umwelt‑, Klima- und Energiegerechtigkeitsdebatte besonders wichtig (Schlosberg 2007, S. 112 ff.). Erstens bezieht sich Verteilungsgerechtigkeit immer auch auf die (global) ungleiche Nutzung von Senken^3^ (vor dem Hintergrund der historischen ökologischen Schuld der Länder des globalen Nordens, die die Hauptverursacher des Klimawandels sind). Gleiches gilt zweitens für die (global) ungleiche Verteilung sowohl der schädlichen Folgen von Klimaschutzpolitik (in Bezug auf die Ausbeutung von Arbeit und Natur bei der Gewinnung von Ressourcen und der Produktion von Technologien und Infrastrukturen sowie in Bezug auf deren Installation) als auch der Klimakrise selbst (die tendenziell die Länder des globalen Südens besonders hart trifft). Drittens spielt die Generationengerechtigkeit eine wichtige Rolle im Hinblick auf die negativen Effekte, die die Emissionen heutiger Generationen auf die Lebensbedingungen zukünftiger Generationen haben werden.

Diese klimaspezifischen Ansprüche werden von der Klimagerechtigkeitsbewegung in Deutschland prominent artikuliert (Sander 2016). Zusätzlich zu ihrer Kernforderung eines schnellen Kohleausstiegs betont die Klimagerechtigkeitsbewegung stets weitere Gemeinwohlziele wie (globale) Gerechtigkeit, Vertiefung von Demokratie und ein grundsätzlich gewandeltes Verhältnis zur Natur. Mit der Aktionsform *Ende Gelände*, bei der mehrere tausend Menschen Tagebaue, Kohlebagger und/oder Kohlekraftwerke blockieren, gewann die Bewegung an Zulauf und öffentliche Aufmerksamkeit (Sander 2016, S. 25 ff., 2017). Einen weiteren Schub erlebte die Klimagerechtigkeitsbewegung durch die rasante Entwicklung der Schulstreiks von *Fridays for Future *(FFF). In kürzester Zeit wurden FFF-Ortsgruppen gegründet und – in Zusammenarbeit mit etablierten NGOs – Massendemonstrationen organisiert, an denen sehr viele Menschen teilnahmen (am 20.09.2019 deutschlandweit 1,4 Mio.) (Rucht und Rink 2020, S. 98). Insgesamt gewann die Klimagerechtigkeitsbewegung mit FFF eine breite Unterstützung von vielen Organisationen und weiten Teilen der Bevölkerung (Koos und Lauth 2020). Im Gegensatz zu *Ende Gelände *gibt es bei FFF keinen kapitalismuskritischen Konsens, auch der Fokus auf globale Gerechtigkeit ist bei FFF schwächer ausgeprägt. Die ambivalente Positionierung gegenüber *Ende Gelände *(und den mit der Kampagne transportierten radikaleren Forderungen) entwickelte sich mit der Zeit in Richtung einer vorsichtigen Annäherung und gegenseitiger solidarischen Bezugnahme (Teune 2020). Insgesamt bleibt das Spektrum an politischen Positionen innerhalb von FFF aber vergleichsweise breit, den gemeinsamen Nenner bildet die Forderung nach konsequentem Klimaschutz.

Neben der primär kampagnenförmig agierenden Klimagerechtigkeitsbewegung machten die großen, überregional agierenden Umwelt-NGOs und die lokalen Bürger*inneninitiativen mit ihrer stetigen Arbeit (von juristischen Auseinandersetzungen über die Einbringung in Planungs- und Genehmigungsverfahren bis hin zu Informationskampagnen und Protestaktionen) die Kohleverstromung zu einem besonders virulenten Konfliktfeld. Obwohl die von der Bundesregierung eingesetzte *Kommission für Wachstum, Strukturwandel und Beschäftigung* („Kohlekommission“) Anfang 2019 ihren Abschlussbericht nahezu einstimmig verabschiedete, bleibt das Thema weiterhin kontrovers. Mit den im Juli 2020 verabschiedeten *Kohleausstiegs-* und *Strukturstärkungsgesetzen *zeigten sich zwar die Befürworter*innen eines eher langsamen Kohleausstiegs (Landesregierungen der Braunkohlereviere, Betreibergesellschaften der Tagebaue und Kraftwerke sowie die Gewerkschaften Deutscher Gewerkschaftsbund DGB, Vereinte Dienstleistungsgewerkschaft ver.di und Industriegewerkschaft Bergbau, Chemie, Energie IG BCE) tendenziell zufrieden. Dagegen übten Akteur*innen, die primär klimapolitisch argumentieren (Umwelt-NGOs, Klimaaktivist*innen, Wissenschaftler*innen) scharfe Kritik, weil mit dem geplanten Kohleausstieg die Ziele des Pariser Klimaabkommens nicht erreicht werden können (Oei et al. 2020, S. 3). Kritisiert wird unter anderem, dass 2020 mit *Datteln 4* ein weiteres Steinkohlekraftwerk in Betrieb genommen wurde und dass die Braunkohlekraftwerke nicht, wie von der *Kohlekommission* gefordert, gleichmäßig vom Netz gehen, sondern jeweils gehäuft in den Jahren 2029 und 2038, was die Emissionen erhöht (ebd., S. 6 ff.). Weiterhin werden die Entschädigungszahlungen von insgesamt 4,35 Mrd. € für die Betreibergesellschaften als unrechtmäßig und zu hoch kritisiert (ebd., S. 6 f.). Der Kohleausstieg könnte laut einem Statement von *Scientists for Future „*kostengünstiger und effektiver gestaltet werden“ (Scientists for Future 2020). Tatsächlich besteht – angesichts der verschlechterten Wettbewerbslage der Kohleverstromung – die Gefahr, dass aufgrund der hohen Entschädigungszahlungen Kohlekraftwerke länger betrieben werden als es ohne das Gesetz erfolgt wäre. Schließlich wird eine Entschädigung nur unter der Voraussetzung gewährt, dass die Kraftwerke bis zum vereinbarten Abschalttermin Strom produziert haben. Aus betriebswirtschaftlicher Perspektive kann es demnach plausibel sein, Kohlekraftwerke trotz Unrentabilität weiter zu betreiben, sofern die in Aussicht gestellten Entschädigungszahlungen die Verluste mehr als ausgleichen.

Die Klimagerechtigkeitsbewegung und die Umwelt-NGOs fokussierten in den letzten Jahren auf den Kohleausstieg als nächstes Etappenziel der Energiewende. Mittlerweile wenden sich aber immer mehr zivilgesellschaftliche Akteur*innen dem Thema der (ausbleibenden) Mobilitätswende zu. Klassischerweise gab und gibt es Proteste gegen den Bau von Autobahnen oder Bundesstraßen. Neben dieser Kritik an einzelnen Infrastrukturprojekten gibt es – und das ist das Besondere an der neuen Protestwelle – vermehrt prinzipielle Kritik an (der Privilegierung von) Automobilität und Flugverkehr. Zum einen gibt es verstärkt lokale (Bürger*innen‑)Initiativen wie den *Münchner Radentscheid*, den *Radentscheid Aachen*, den *Volksentscheid Fahrrad* in Berlin oder die *Volksinitiative Aufbruch Fahrrad *in NRW, die sich für eine Neuordnung des innerstädtischen Verkehrs engagieren. Zum anderen entwickelten sich in den letzten Jahren Akteur*innen bzw. Kampagnen wie *Am Boden bleiben, Sand im Getriebe *und *attac* (Kampagne *einfach.umsteigen: Klimagerechte Mobilität für alle!*), die sich innerhalb der Klimagerechtigkeitsbewegung verorten und für eine drastische Reduktion des motorisierten Individualverkehrs bzw. des Flugverkehrs einsetzen. Diese neuen Formen des Protestes verbinden sich im Fall des „Danni“ (Dannenröder Wald) mit dem klassischen Widerstand gegen ein konkretes Infrastrukturprojekt. Im politischen Engagement der letzten Jahre gegen den Weiterbau der A 49 und für den Erhalt des Dannenröder Waldes werden die umfassenden Forderungen nach einer Mobilitätswende mit dem konkreten Anliegen der Verhinderung einer Autobahn-Strecke verknüpft. Was die Symbolik und das Aktionsrepertoire (z. B. Baumbesetzungen) betrifft, gibt es explizite Bezüge zu den die Anti-Kohle-Protesten gegen die Erweiterung des Braunkohle-Tagebaus Garzweiler und für den Erhalt des „Hambi“, des Hambacher Waldes. Erfolgreiche Mobilisierungsstrategien aus den Anti-Kohle-Protesten werden somit auf das Handlungsfeld der Mobilitätswende übertragen. Trotz der gestiegenen Aufmerksamkeit ist es angesichts der ausbleibenden Transformationserfolge weiterhin gerechtfertigt, Mobilität als blinden Fleck der Energiewende zu bezeichnen (Canzler und Wittowsky 2016). Schließlich ist Verkehr der einzige Sektor in Deutschland, in dem im Vergleich zu 1990 die Treibhausgasemissionen nicht zurückgegangen, sondern weiter angestiegen sind (Umweltbundesamt 2019, S. 71).

Bis hierhin habe ich vor allem Konflikte um Exnovationen betrachtet, d. h. Konflikte um fossil-nukleare Energieinfrastrukturen, die im Namen der Klimagerechtigkeit rückgebaut werden sollen, wogegen sich diejenigen wehren, die vom bestehenden Energieregime bislang profitiert haben (zu Konflikten um Exnovationen vgl. David und Gross 2019; Krüger und Pellicer-Sifres 2020). Daneben gibt es aber auch Verteilungskonflikte um den Ausbau der erneuerbaren Energien. In Studien zu diesem Themenkomplex wird grundsätzlich davon ausgegangen, dass die Chance, an den ökonomischen Vorteilen der geplanten Energieprojekte teilhaben zu können, bei den Anwohner*innen maßgeblich zu einer positiven Haltung gegenüber selbigen beitragen würde (Wolsink 2007). Allerdings merkt Eichenauer (2018, S. 328f.) an, dass die Möglichkeit der finanziellen Beteiligung von betroffenen Kommunen oder Anwohner*innen je nach Ausgestaltung unterschiedliche Wirkung entfaltet. Eine frühzeitig angelegte finanzielle Beteiligung kann durchaus zu mehr Akzeptanz führen. Allerdings kann eine in Aussicht gestellte finanzielle Beteiligung auch zu stärkerer Ablehnung führen, sofern diese Option erst nach Ausbruch eines lokalen Konfliktes ins Spiel gebracht wird und als Bestechung empfunden wird.

Das Verhältnis von Stadt und Land ist ein besonders brisanter Punkt in den Konflikten um Verteilungsgerechtigkeit. Ein Argument von Windkraftkritiker*innen lautet, dass über die Energiewende in den Städten entschieden werde, die Landbevölkerung aber die Kosten tragen müsse (Messinger-Zimmer und Zilles 2016, S. 49). Ländliche Gegenden sind überproportional für die Energieproduktion verantwortlich und Städte haben einen hohen Energiebedarf (Quitzow et al. 2016, S. 167). Diese Asymmetrie verschärft sich, wenn die ländlichen Gegenden der Energieproduktion, die unter den damit einhergehenden (gesundheitlichen, landschaftsästhetischen usw.) Beeinträchtigungen leiden, nicht (angemessen) an den Profiten beteiligt werden (Gailing und Röhring 2015, S. 40). Wenn die kommunale und regionale Wertschöpfung begrenzt ist, weil in erster Linie regionsexterne Investor*innen profitieren, werden ländliche Räume nicht zu aktiven Gestaltungsräumen, sondern zu passiven Installationsräumen, „denen die Aufgabe der Energieproduktion durch externe Planungen und Investitionsentscheidungen „übergeholfen“ wird“ (ebd., S. 37).

Ländliche Energieregionen entwickeln sich allerdings sehr divers, wie Gailing und Röhring (2016) durch einen Vergleich zwischen Prignitz und Lüchow-Dannenberg zeigen. Während es in Lüchow-Dannenberg durch die Anti-Atom-Bewegung historisch gewachsene kollektive Organisationsstrukturen gibt, die sich aktiv für die Energiewende einsetzen und diese gestalten wollen und können, fehlen in der Prignitz derartige Ressourcen (ebd., S. 243 f.). In der Prignitz investierten viele regionsexterne Unternehmen in die Produktion erneuerbarer Energien. Lokale Akteur*innen fühlten sich übergangen (ebd.). In der Folge ist die Akzeptanz für die Energiewendeprojekte gering, so dass die großen Windparks als Ergebnis einer „Kolonisierung“ der Region durch externe Investor*innen wahrgenommen werden (ebd., S. 243). In Lüchow-Dannenberg konnte dagegen gezeigt werden, dass die Energiewende auch das Potenzial für ein regionales *Empowerment* bietet (ebd., S. 244). Für diese positive Entwicklung war das Engagement und die Ressourcen von einzelnen Schlüsselfiguren zentral, insbesondere von lokalen Landwirt*innen, welche die Energieproduktion in die eigenen Hände nahmen (ebd.). Mit Unterfranken hat Galvin (2018) wiederum eine Region analysiert, in der die Wahrnehmung und Gestaltung der Energiewende einem starken Wandel unterlag. So wurde die Energiewende zunächst von vielen Akteur*innen als Chance für regionale Wertschöpfung begriffen. Energiegenossenschaften trieben einen dezentralen Ausbau von erneuerbaren Energien voran (ebd., S. 270). Jedoch hat der Wandel des EEG – von der festen Einspeisevergütung zu einem Auktionsverfahren – die Gestaltungsmacht zugunsten von Investor*innen mit hohem Kapitaleinsatz verschoben. Diese sind automatisch im Vorteil, da sie niedrigere Renditen bei höherem Risiko tolerieren können (ebd.). Viele lokale Akteur*innen sehen sich zunehmend einer Allianz aus Politiker*innen, Konzernen und externen Investor*innen ausgeliefert (ebd., S. 272 ff.). Sie kritisieren nicht nur die fehlende regionale Wertschöpfung, für sie ist die Energiewende als Ganzes diskreditiert (ebd., S. 275).

Der Aspekt von räumlich ungleich verteilten Kosten und Nutzen der Energiewende spielt auch in den Auseinandersetzungen um den Ausbau der Stromtrassen eine wichtige Rolle. Hier wird kritisiert, dass vornehmlich die Regionen am Anfangs- bzw. Endpunkt profitieren würden, in denen entweder viel Strom produziert oder abgenommen werde. Die Trassenmitte hätte dagegen keine Vorteile, müsse aber unter dem Trassenbau und den damit verbundenen befürchteten landschaftsästhetischen und gesundheitlichen Beeinträchtigungen leiden (Messinger-Zimmer und Zilles 2016, S. 49; Neukirch 2016).

In diesem Zusammenhang wurde die NIMBY-Debatte (benannt nach der Maxime „Not In My Back Yard“, der englischsprachigen Fassung des St.-Florians-Prinzips) in Politik und Wissenschaft sehr intensiv geführt. In der Debatte geht es um die Frage, inwiefern es gerechtfertigt ist, lokale Proteste (insbesondere gegen Windkraftanlagen und Stromtrassen) in erster Linie nach der Logik des NIMBY-Ansatzes zu beschreiben (Becker et al. 2016, S. 44). Der NIMBY-Ansatz geht davon aus, dass lokale Anwohner*innen zwar prinzipiell die Energiewende befürworten, geplante Projektvorhaben in ihrer unmittelbaren Umgebung jedoch aus egoistischen Gründen ablehnen würden. Diese These wurde von vielen Autor*innen stark kritisiert, da sie eine Verkürzung der komplexen Motivationslage von Protestgruppen darstelle und deren Kritikpunkte nicht ernst nehme (Eichenauer 2018, S. 317; Reusswig et al. 2016, S. 222; van der Horst 2007, S. 2713; Wolsink 2007).

Von dieser Einschätzung unbenommen bleibt die Tatsache, dass lokale Konflikte um den Zubau von Windenergie, Biomasseanlagen und Stromtrassen zunehmen (Messinger-Zimmer und Zilles 2016, S. 42; Reusswig et al. 2016, S. 225). Dabei beanspruchen stets alle Konfliktparteien für sich, Gemeinwohlinteressen zu vertreten (Messinger-Zimmer und Zilles 2016, S. 45). So argumentieren beispielsweise die Befürworter*innen von Windenergie mit Klimaschutz, Verringerung der Abhängigkeit von Energieimporten, regionaler Wertschöpfung und der Schaffung von Arbeitsplätzen, wohingegen die Gegner*innen die Verschandelung der Landschaft, mögliche Gesundheitsgefahren durch Geräuschemissionen sowie die Beeinträchtigung für Tourismus und Naturschutz anführen (Becker et al. 2016, S. 42; Gailing und Röhring 2015, S. 39). Gleichzeitig spielt die persönliche Betroffenheit durchaus eine wichtige Rolle. Schließlich sind die befürchteten Einbußen an Lebensqualität, die Beeinträchtigungen durch Verschattung sowie der Wertverlust von Grundstücken ebenfalls zentrale Argumente der Windkraftkritiker*innen (Eichenauer 2018, S. 328). Es bleibt stets eine Frage der Perspektive, inwieweit die Sorge um die nähere Umgebung als NIMBY-Attitüde oder als Sorge um das Gemeinwohl interpretiert wird. Zum besseren Verständnis der vielschichtigen Gemengelage lohnt in dem Zusammenhang der Blick auf den Forschungsstrang zu Fragen von *place identity* bzw. Heimatverbundenheit in Energiekonflikten (van der Horst 2007, S. 2709). Dabei geht es nicht nur um Fragen der Verteilung von Kosten und Nutzen, sondern auch um den Anspruch auf die Aufrechterhaltung bestimmter (lokaler) Identitäten und Lebensqualitäten, die je nach Perspektive als legitimer Anspruch oder als unhaltbares Privileg bezeichnet werden. Für die Analyse dieser Konstellationen kann das Konzept der Anerkennungsgerechtigkeit hilfreich sein.

### Konflikte um Anerkennungsgerechtigkeit

Das Ziel der Anerkennungsgerechtigkeit adressiert systematische Unterschiede in der Repräsentation von Gruppen bzw. asymmetrische gruppenspezifische Möglichkeiten, Deutungshoheit zu erlangen (Fraser 2003, S. 13). Der Fokus liegt hier auf Fragen der Gerechtigkeit, die sich aus der Statusordnung der Gesellschaft ergeben. Anerkennungsgerechtigkeit, wie Fraser sie versteht, zielt letztlich auf „a difference-friendly world, where assimilation to majority or dominant cultural norms is no longer the price of equal respect“ (Fraser 2003, S. 7). Im Fall der Energiewende geht es um Fragen der Wertschätzung, Repräsentation und Deutungshoheit von (lokalen) Identitäten und gruppenspezifischen Energiewende-Visionen.

Der Widerstand gegen bestimmte Energieprojekte wird oftmals als Anspruch auf den Erhalt der eigenen Heimat artikuliert. Der Begriff „Heimat“ spielt für viele lokale Aktivist*innen eine wichtige Rolle. Sie sehen ihr Engagement als Einsatz für die Bewahrung eines lebenswerten Umfeldes, was – anders als der NIMBY-Begriff suggeriert – über die Verteidigung von eigenem Haus und Grund hinaus geht (Marg et al. 2013, S. 103 f.). Dieses Engagement basiert in der Regel auf einer regen Zivilgesellschaft, ressourcenstarken Bürger*innen, einer lokalen Identifikation und einer hohen Selbstwirksamkeitserwartung (Galvin 2018, S. 274; Marg et al. 2013, S. 103 f.; Marg 2017, S. 230). Marg (2017, S. 225) spricht von Heimat als Kampfbegriff, der eine Reaktion auf die durch die Globalisierung ausgelösten Prozesse des *disembedding*^4^ darstelle. Die Suche nach Heimat sieht sie somit auch als „Ausdruck des Strebens nach Entkomplexisierung und des Umgehens von Kontingenz“ (Marg 2017, S. 227). Marg betont, dass die Referenz auf Heimat nicht zwangsläufig mit Traditionalismus, Konservatismus und Antimodernität gleichzusetzen sei, sondern auch verstanden werden müsse als Ressource für eine aktive Zivilgesellschaft, die den Wunsch und die Fähigkeit nach (Mit‑)Gestaltung der eigenen Lebensumstände entwickelt (Marg 2017, S. 229 f.). Gleichzeitig verweist sie auf Erscheinungsformen, in denen exkludierende, chauvinistische Strategien vorherrschen (Marg 2017, S. 231). Was alle Spielarten der politischen Artikulation von Heimat gemeinsam haben ist, dass sie der Wahrnehmung entspringen, die anstehenden Veränderungen würden einen rücksichtslosen Eingriff von außen darstellen und die eigenen Einflussmöglichkeiten müssten erst erkämpft werden (Marg 2017, S. 229). In der Debatte um Heimat wird somit das Spannungsfeld zwischen externen Projektplaner*innen und Entscheidungsträger*innen auf der einen Seite und den Anwohner*innen auf der anderen Seite verhandelt.

Ein Hauptargument für die Skepsis gegenüber Projektplaner*innen und Entscheidungsträger*innen ist die wahrgenommene Unglaubwürdigkeit der von ihnen zur Verfügung gestellten Informationen und Gutachten, die als einseitig kritisiert werden (Eichenauer 2018, S. 330 ff.). Tatsächlich genießen – auch bei unbeteiligten, aber betroffenen Bürger*innen – gerade jene Akteur*innen das geringste Vertrauen, denen der größte Einfluss zugesprochen wird und umgekehrt (Hanisch und Messinger-Zimmer 2017, S. 172 f.). Den Unternehmen und den höheren Ebenen der Politik wird der größte Einfluss zugesprochen, gleichzeitig genießen sie das geringste Vertrauen. Tatsächlich vertreten fühlen sich die betroffenen, aber nicht aktiven, Bürger*innen am ehesten von lokalen Bürger*inneninitiativen, die sie als ihre Interessenvertretung wahrnehmen (Hanisch und Messinger-Zimmer 2017, S. 174; Messinger-Zimmer und Zilles 2016, S. 47). Die Kritik von Bürger*innen und zivilgesellschaftlichen Initiativen an der Planung und Durchführung von Energiewende-Projekten mündet in den Zweifeln, inwieweit die Meinung der Betroffenen für die Entscheidungsträger*innen überhaupt eine relevante Größe darstelle (Hanisch und Messinger-Zimmer 2017, S. 175). In Bezug auf die damit angesprochenen Fragen der Anerkennungsgerechtigkeit konstatieren empirische Studien „ein tief sitzendes Misstrauen der Betroffenen gegenüber Planern aus Politik, Verwaltung und Unternehmen, die die Interessen der betroffenen Bevölkerung und die negativen Folgen für die Umgebung eben nicht in die Definition des Gemeinwohls einkalkulieren würden“ (Messinger-Zimmer und Zilles 2016, S. 49; vgl. auch Eichenauer 2018, S. 336). Diese Phänomene verweisen auf eine Repräsentationskrise, die sich nicht nur, aber eben auch in besonderer Weise im Handlungsfeld der Energiewende zeigt. In meiner Interpretation bilden sie die erste Dimension der Demokratiekrise.

Die AfD knüpft in ihrer Mobilisierung gegen Energiewendeprojekte (insbesondere gegen Windkraftanlagen) an die Repräsentationskrise an und verschärft sie zugleich. Die AfD erhält verstärkt Zuspruch von Akteur*innen, die sich in Konflikten um konkrete Energiewendeprojekte nicht gehört fühlen, weil sie von den (ihrer Wahrnehmung nach) geringen Möglichkeiten politischer Teilhabe und Mitbestimmung enttäuscht sind (Eichenauer et al. 2018, S. 639ff.). Zugleich distanzieren sich auch viele Windkraftkritiker*innen vehement von der Partei (ebd., S. 641). Die AfD koppelt die Kritik an der Energiewende mit ihrer Ablehnung pluralistisch-demokratischer Institutionen (z. B. Minderheitenrechte und Pressefreiheit), was sich auch daran zeigt, dass sie ein instrumentelles Verständnis von Bürger*innenbeteiligung hat und sich nicht ernsthaft für eine Ausweitung demokratischer Aushandlungsprozesse einsetzt (ebd., S. 641 f.). Die Verknüpfung der Kritik an der (Umsetzung der) Energiewende mit rechtsautoritären bis rechtsextremen Positionen, die pluralistisch-demokratische Institutionen ablehnen, stellt die zweite Dimension der Demokratiekrise dar.

Die Auseinandersetzungen um Fragen von Anerkennungsgerechtigkeit verweisen auf eine neue Konfliktkonstellation, in der erneuerbare Energien nicht mehr per se als *Underdog*, als David, sondern zunehmend auch als Ausdruck von Macht, als Goliath, wahrgenommen werden (Mautz 2010, S. 185 f.). Die erneuerbaren Energien galten lange Zeit als unbefleckte Alternative zum übermächtigen atomar-fossilistischen Regime und dessen zerstörerische Effekte auf Menschen und Natur. Mit der Expansion von erneuerbaren Energien, dem größer werdenden Anteil an zentralisierten Großprojekten und dem gestiegenen ökonomischen Erfolg wurden sie allerdings zunehmend selbst als Gefahrenquelle für Menschen und Natur gesehen (Mautz 2010, S. 185 f.; Ohlhorst und Schön 2010, S. 216 f.).^5^ Diese Verschiebung der Wahrnehmung betrifft besonders den Windkraftsektor, der durch eine „zunehmende räumliche Konzentration und Verbraucherferne, eine zunehmende Professionalisierung, eine immer weitergehende Arbeitsteilung sowie zunehmend anonyme Beziehungen zwischen einer steigenden Anzahl beteiligter Akteure“ (Ohlhorst und Schön 2010, S. 217) gekennzeichnet ist. Mit dem Wandel der Konfliktkonstellationen änderten sich auch die Strategien und Organisationsformen der Windkraftkritiker*innen. Bürger*inneninitiativen gegen Windkraftanlagen professionalisieren sich und es entstehen überregionale Organisationen (Eichenauer 2018, S. 320). Damit steigen die finanziellen Ressourcen, das Know-how und der politische Einfluss. Die Konflikte um Windkraft nehmen in der öffentlichen Debatte mehr Raum ein und der Bau von Windkraftanlagen entwickelte sich zu einem Wahlkampfthema, insbesondere in ländlichen Gegenden, wie man beispielsweise an den Landtagswahlen 2019 in Brandenburg, Sachsen und Thüringen beobachten konnte.

### Konflikte um Verfahrensgerechtigkeit

Das Ziel der Verfahrensgerechtigkeit formuliert Fraser (2003, S. 36) als Parität der Teilhabe. Nach Fraser kann es echte Parität der Teilhabe nur geben, wenn es Verteilungs- und Anerkennungsgerechtigkeit gibt (Fraser 2003, S. 39 f.). Umgekehrt wird Verteilungs- und Anerkennungsgerechtigkeit nur erreicht, wenn es faire, demokratische Institutionen und Verfahren zur Entscheidungsfindung gibt. Die zirkuläre Annäherung an die Gerechtigkeit kann nur durch öffentliche demokratische Verhandlung erreicht werden (Fraser 2003, S. 43). Fraser will diesen öffentlichen Aushandlungsprozessen nicht mit vermeintlich theoretisch fundierten konkreten Vorschlägen zuvorkommen. Sie formuliert jedoch ein abstraktes Kriterium zur Unterscheidung von legitimen und nicht-legitimen Ansprüchen auf Gerechtigkeit: Anspruchsteller*innen müssen zeigen, dass die gegenwärtigen Arrangements sie daran hindern, gleichberechtigt am gesellschaftlichen Leben teilzunehmen (Fraser 2003, S. 38). Im Hinblick auf die Energiewende ist hier die zentrale Frage, inwieweit es nicht nur formale Beteiligungsrechte, sondern auch tatsächliche Einflussmöglichkeiten gibt – für wen und in welchem Maße?

Dieser Aspekt wird beispielsweise in Konflikten um den Ausbau der Stromnetze relevant, wenn lokale Akteur*innen aus betroffenen Gegenden die Annahmen hinterfragen, auf deren Basis der Ausbaubedarf von der Bundesregierung festgelegt wurde (Galvin 2018, S. 276). Ein Problem ist dabei, dass die Forderung nach einer dezentralen, verbrauchernahen Energieversorgung, die (insbesondere in Kombination mit Suffizienzstrategien) den Bedarf an neuen Stromtrassen reduzieren würde, in Beteiligungsverfahren nicht eingebracht werden kann (Barry und Ellis 2011). Darüber hinaus stellt die wahrgenommene Intransparenz der Verfahren einen zentralen Kritikpunkt dar. Die Kritik der zivilgesellschaftliche Akteur*innen, die sich gegen den Ausbau von Stromtrassen einsetzen, kulminieren regelmäßig in der artikulierten Wahrnehmung, dass sie letztlich keine Einflussmöglichkeiten auf Planungsprozesse haben (Galvin 2018, S. 272; Messinger-Zimmer und Zilles 2016, S. 44).

Das Bedürfnis nach (Wieder‑)Erlangung von lokal verankerter Entscheidungskompetenz und demokratischer Mitsprache zeigt sich ebenfalls in der Rekommunalisierungs-Welle (Becker et al. 2016, S. 41). Die Versuche der Rekommunalisierung von Stadtwerken und Energienetzen gehen in der Regel mit starken Konflikten einher, in denen zivilgesellschaftliche Akteur*innen oftmals auf das Mittel von Bürger*innen- und Volksbegehren zurückgreifen (Haas und Sander 2018, S. 352). Die Neugründungen von Stadtwerken (72 Neugründungen im Energiebereich zwischen 2005 und 2012) bietet Chancen für eine Demokratisierung der Energiewende und für einen größeren Einfluss der Lokalpolitik auf ihre Ausgestaltung (Haas und Sander 2018; Wagner und Berlo 2017). In dem Zusammenhang sollte allerdings beachtet werden, dass die Einflussmöglichkeiten von Bürger*innen auf die Stadtwerke erheblich variieren, was unter anderem auf unterschiedliche Unternehmensstrukturen zurückzuführen ist (Haas und Sander 2018, S. 351). So sind manche Stadtwerke auch als Aktiengesellschaft (AG) oder Gesellschaft mit begrenzter Haftung (GmbH) organisiert. Dies hat zur Folge, dass der demokratische Souverän zwar formal der Eigentümer ist, faktisch aber oft nur die Spitzen der Exekutive an Entscheidungen beteiligt sind, wohingegen weder Kommunalabgeordnete noch einfache Bürger*innen Einfluss auf die Geschäftspolitik nehmen können (ebd.). Insofern werden Stadtwerke oftmals wie private Unternehmen geführt und sind auf Profitmaximierung ausgerichtet. Deshalb plädieren Haas und Sander dafür, dass die etwa 900 Stadtwerke, die es in Deutschland gibt, selbst demokratisiert werden, „damit Möglichkeiten der Einflussnahme durch die Bevölkerung erweitert oder überhaupt erst geschaffen werden“ (ebd.).

Insgesamt gilt auch für die Verfahrensgerechtigkeit, was bereits für die Verteilungsgerechtigkeit festgestellt wurde, nämlich dass die konkrete Ausgestaltung der Beteiligung einen großen Unterschied für die Einstellung der Anwohner*innen zu geplanten Energieprojekten macht (Eichenauer 2018, S. 329). Die Stärkung von Partizipationsmöglichkeiten führt nicht automatisch zu einer höheren Zustimmung zu Energiewende-Projekten. Entscheidend ist, inwieweit die von den Anwohner*innen vorgebrachten Argumente in institutionalisierten Verfahren ernsthaft geprüft werden. Da diese Punkte aus Sicht der Protestierenden regelmäßig missachtet werden, kritisieren Bürger*inneninitiativen zunehmend die Genehmigungs- und Beteiligungsverfahren als solche (Eichenauer 2018, S. 336). Diese Phänomene interpretiere ich als Legitimationskrise (Radtke 2020, S. 99) und damit als dritte Dimension der Demokratiekrise.

## Demokratietheoretische Reflexion der Gemeinwohlkonflikte in der Energiewende

Im vorangegangenen Kapitel wurde herausgearbeitet, dass die Auseinandersetzung um die Definition und Priorisierung von Gemeinwohlzielen der Energiewende in den Konflikten um Politikinstrumente und um konkrete Energieprojekte ausgetragen werden. Im Folgenden wird begründet, warum eine einseitige Orientierung am Paradigma der deliberativen Demokratie die konstatierten Tendenzen einer Demokratiekrise weiter verschärft (2.1). Diese Kritik aufgreifend, wird ein radikaldemokratisches Verständnis von Gemeinwohlkonflikten entwickelt und radikaldemokratische Vorschläge für die produktive Bearbeitung von Energiekonflikten erörtert (2.2).

### Energiekonflikte und die Grenzen der Deliberation

Für die Planung und Umsetzung von Energieprojekten gilt das deliberative Modell, das auf demokratietheoretische Überlegungen von Habermas zurückgeht, als normativer Orientierungspunkt (Barry und Ellis 2011, S. 31 f.; Kersting und Roth 2018, S. 1153). In deliberativen Verfahren steht die Teilnahme von Bürger*innen an öffentlichen Debatten und Entscheidungsfindungsprozessen im Zentrum. Dabei ist entscheidend, dass alle Positionen in die Debatten und Beteiligungsverfahren eingebracht werden können und dass allein die Rationalität der Argumente zählt. Die unterschiedlichen Machtressourcen der beteiligten Akteur*innen sollen keinen Einfluss haben. Habermas spricht von einer „idealen Sprechsituation“ (Habermas 1973, S. 152), an der sich reale Entscheidungsprozesse messen lassen sollen. Er hält daran fest, dass es grundsätzlich möglich sei „kraft Argumentation die jeweils verallgemeinerungsfähigen Interessen von denen zu scheiden, die partikular sind und bleiben“ (ebd., S. 149). Sich widerstreitende Positionen sollen unter dem Gesichtspunkt der Verallgemeinerbarkeit einer diskursiven Prüfung unterzogen werden, so dass im Konsens das Gemeinwohl ermittelt werden kann (Habermas 1997). Der deliberative Ansatz entwickelte sich zur einflussreichsten Demokratietheorie und wurde „in einer Vielzahl von deliberativen Foren, Versammlungen, etc. empirisch implementiert“ (Radtke und Schaal 2018, S. 148). Die Bedeutung des deliberativen Modells als normativer Orientierungspunkt für partizipative Leitbilder und Beteiligungsleitlinien gilt insbesondere auch für die Planung und Realisierung von Energieinfrastrukturprojekten (Barry und Ellis 2011, S. 31 f.; Kersting und Roth 2018, S. 1158). Allerdings entspricht die Planung und Durchführung von Energieprojekten den hohen Ansprüchen des deliberativen Demokratiemodells oftmals nur ungenügend (Barry und Ellis 2011, S. 33; Huge und Roßnagel 2018; Kersting und Roth 2018, S. 1156 f.). So wurde (nicht nur) für das Handlungsfeld der Energiewende nachgewiesen, dass in deliberativen Prozessen finanz- und bildungsstarke Milieus dominieren (Fraune 2018, S. 760f.; Radtke und Schaal 2018, S. 150f). Im Folgenden argumentiere ich, dass es sich bei den Mängeln nicht allein um (behebbare) Umsetzungsfehler handelt, sondern dass das deliberative Modell auf wenig plausiblen Annahmen basiert, und dass eine Orientierung an der „idealen Sprechsituation“ Tendenzen einer Demokratiekrise verstärken kann.

Deliberative Verfahren haben sowohl auf der Ebene der beteiligten Personen als auch auf der Ebene der verhandelten Inhalte notwendigerweise exkludierende Effekte. Trotz Abwesenheit formaler Zugangsschranken und trotz Zusicherung formal gleicher Mitspracherechte ist der Versuch der Ausklammerung sozialer Ungleichheiten zum Scheitern verurteilt, weil sich die Machtasymmetrien sowohl in die gesellschaftlichen Normen und Institutionen als auch in die Alltagspraxen und Identitäten eingeschrieben haben, aus denen heraus eine allgemeine Geltung für bestimmte Positionen postuliert wird (Fraser 1990, S. 62ff.). Aufgrund dieser indirekten Machteffekte wirkt sich die kontrafaktische Orientierung am Ideal der Machtfreiheit in der Regel zum Nachteil marginalisierter und zum Vorteil privilegierter Gruppen aus, deren Forderungen sich in die hegemoniale Weltanschauung einfügen (ebd., S. 64). Darüber hinaus setzen deliberative Verfahren eine bestimmte kommunikative Kompetenz voraus (Huge und Roßnagel 2018, S. 616f) und verschaffen somit jenen Personengruppen mehr Gehör, die ohnehin schon über die besseren Einflussmöglichkeiten verfügen (Fraune 2018, S. 760 f.; Radtke und Schaal 2018, S. 150 f.).

Was die Ebene der Themen betrifft, lautet ein zentraler Kritikpunkt an deliberativen Verfahren, dass nur vorab definierte Inhalte verhandelt werden können (Barry und Ellis 2011, S. 32). Deliberative Entscheidungsfindungsprozesse sind auf die stabilen Rahmenbedingungen eines etablierten Institutionengefüges angewiesen und auch nicht darauf ausgelegt, diese grundlegenden Parameter zu verschieben (Nonhoff 2019, S. 296 ff.). In einem ambitionierten Beteiligungsverfahren könnte beispielsweise darüber verhandelt werden, mit welcher monetären Beteiligung der betroffenen Kommune(n) ein konkretes Energieinfrastrukturprojekt umgesetzt wird. Darüber hinausgehende Fragen, welche die gesamtgesellschaftliche Transformationsstrategie oder übergreifende Ungleichheitsdimensionen betreffen, haben darin keinen Platz (Barry und Ellis 2011, S. 32). Dabei sind dies berechtigte Fragen, die auch die konkret zu treffende Entscheidung tangieren bzw. unter Umständen zu Antworten führen können, welche die ursprüngliche Frage nach der adäquaten Umsetzung eines Projekts erübrigen würden (z. B. im Falle einer konsequenten Stärkung von Suffizienzstrategien und der daraus folgenden Senkung des Bedarfs an Energieinfrastrukturprojekten). Dieses Problem besteht teilweise aufgrund des Zuschnitts von Zuständigkeitsbereichen im politischen Mehrebenensystem, d. h. deliberative Prozesse auf kommunaler oder regionaler Ebene können nur über Entscheidungen beraten, die auch tatsächlich auf diesen Ebenen entschieden werden können (und über die nicht bereits auf höheren Ebenen entschieden wurde). Eine Stärkung von deliberativen Prozessen auf höheren Ebenen – im genannten Beispiel in Bezug auf die Energiewende-Strategie auf nationaler oder europäischer Ebene – könnte das Problem evtl. abschwächen, aber nicht vollends auflösen. Davon abgesehen, dass deliberative Verfahren auf höheren Ebenen äußerst zeit- und kostenintensiv sind, wird es auch dort stets Positionen geben, die nicht in den deliberativen Prozess eingebracht werden können, weil sie auf eine umfassende Neuausrichtung des Gemeinwohls zielen. Dies spricht nicht unbedingt gegen den Einsatz von Beteiligungsverfahren, schließlich ist die monetäre Beteiligung der betroffenen Kommunen – um bei dem Beispiel zu bleiben – nicht unrelevant. Beteiligungsverfahren, die den Anforderungen des deliberativen Demokratiemodells (frühzeitig, transparent, öffentlich, alternativenreich, ergebnisoffen, fair, verbindlich und wirksam, Kersting und Roth 2018, S. 1156f) gerecht werden, können eine durchaus produktive Rolle bei der Planung und Durchführung von konkreten Energiewende-Projekten spielen. Allerdings hat die kontrafaktische Orientierung am Ideal der Machtfreiheit notwendigerweise – und nicht nur bei mangelhafter Umsetzung – exkludierende Effekte.

Darüber hinaus hat die Einhegung von Konflikten durch Konsens-Verfahren problematische Implikationen, weil die Exklusion von Positionen als vernunftbasierte Entscheidung gilt (Mouffe 2015, S. 84). Aus radikaldemokratischer Perspektive steht dem entgegen, dass es keine neutrale Unterscheidung von Gemeinwohl- und Partikularinteressen geben kann (Fraser 1990, S. 70 ff.). Einer solchen Unterscheidung liegen notwendigerweise bestimmte (kollektive) Identitäten und Weltanschauungen zugrunde (Bond 2011, S. 165; Mouffe 2015, S. 100). Es gibt genau so wenig per se verallgemeinerbare Gründe wie es per se partikulare Gründe gibt. Stattdessen werden (Partikular‑)Interessen in hegemonialen Praxen universalisiert, d. h. als Gemeinwohl der Gesellschaft postuliert und durchgesetzt. Aus dieser Perspektive ist die Suggestion einer konfliktfreien Praxis des politischen Zusammenlebens kontraproduktiv für die Zurückdrängung der Tendenzen einer Demokratiekrise. Die Diskrepanz zwischen den Erwartungen an Deliberationen als machtfreie und inklusive Prozesse und den tatsächlich erfahrenen Beteiligungsverfahren kann die Phänomene der Politikverdrossenheit und der Abkehr von pluralistisch-demokratischen Institutionen verstärken.

### Radikaldemokratische Vorschläge für die Bearbeitung von Energiekonflikten

Den Kern der radikalen Demokratietheorie (für einen Überblick über theoretische Vorläufer*innen, Vertreter*innen, Positionen und Kontroversen vgl. Comtesse et al. 2019) bildet die Annahme, dass es keine Letztbegründung für eine bestimmte soziale Ordnung gibt (und auch nicht geben kann) und das politische Zusammenleben somit zwangsläufig konfliktbehaftet ist (Mouffe 2000, S. 13; Nonhoff 2013, S. 323 f.). Demokratische Politik sollte insofern nicht auf die Herstellung eines vermeintlichen Konsenses zielen, sondern die Konfrontation zwischen politischen Gegner*innen, die versuchen, ihre jeweiligen Vorstellungen des Gemeinwohls durchzusetzen, forcieren und in einer Weise organisieren, die mit einem pluralistischen Verständnis von Demokratie kompatibel ist (Mouffe 2000, S. 15). Das radikaldemokratische Argument lautet, dass die Vermeidung von politischer Konfrontation entweder zu Apathie und Politikverdrossenheit führt oder, schlimmer noch, zu anti-pluralistischen Formen kollektiver Identifizierung, die nicht mehr demokratisch eingehegt werden können (Mouffe 2000, S. 16 f.). Dahinter steht die Annahme, dass konkurrierende politische Lager, Möglichkeiten der kollektiven Identifizierung mit politischen Forderungen und Gemeinwohlzielen bieten. Fehlt dies, besteht die Gefahr, dass andere Formen kollektiver Identifizierung (beispielsweise mit einer vermeintlich homogenen und abgrenzbaren Kultur oder Rasse) stärker werden (Mouffe 2000, S. 16). Bezogen auf die Energiewende in Deutschland liegt in dem Zusammenhang das genannte Beispiel der AfD – und ihre Verknüpfung der Kritik an der (Umsetzung der) Energiewende mit rechtsautoritären bis rechtsextremen Positionen, die pluralistisch-demokratische Institutionen ablehnen – nahe.

Aus radikaldemokratischer Perspektive lautet die These, dass die Kombination aus einseitiger Orientierung am deliberativen Demokratiemodell plus fehlender Bereitschaft (von Akteur*innen der Regierungspolitik) zur Austragung von Konflikten um die Definition und Priorisierung von Gemeinwohlzielen der Energiewende die Tendenzen der Demokratiekrise weiter verschärfen. Vor dem Hintergrund der konstatierten Grenzen der Deliberation ist es wichtig anzuerkennen, dass konfliktive Prozesse der öffentlichen Auseinandersetzung und der politischen Entscheidungsfindung einen ähnlich hohen Stellenwert haben sollten. Es bedarf agonistischer Streiträume, in denen auch gegenhegemoniale (marginalisierte) Positionen und Grundsatzfragen artikuliert und ausgehandelt werden können (Machin 2020, S. 164 f.). In dem Zusammenhang muss beachtet werden, dass agonistische Politik, so wie Mouffe (2000, S. 15) sie definiert, die antagonistische Wir/Sie-Unterscheidung in einer Weise artikuliert, die mit einem pluralistischen Verständnis von Demokratie kompatibel ist. Dies impliziert, pluralistisch-demokratische Institutionen (wie Meinungsfreiheit, Minderheitenrechte, Pressefreiheit, Rechtsstaatlichkeit usw.) zu stärken und Antagonist*innen nicht als letztlich zu vernichtende Feind*innen, sondern als legitime Gegner*innen zu verstehen. Dazu bedarf es eines „conflictual consensus“ (ebd., S. 16), d. h. der Anerkennung des pluralistischen Kerns moderner Demokratien und der Legitimität von Konflikten.

In diesem Sinne gilt es, politische Entscheidungen als Positionierung zu explizieren und kollektive Leidenschaften für politische Ziele zu mobilisieren (Mouffe 2000, S. 16 f., 2005, S. 20 ff.). Bezogen auf die Energiewende läge der Schlüssel für eine konstruktive Bearbeitung sowohl der klima- als auch der demokratiepolitischen Herausforderungen darin, die Konfrontation zwischen konkurrierenden Energiewende-Visionen (zentralisiert vs. dezentral, suffizienzorientiert vs. wachstumsorientiert, demokratisch kontrolliert vs. marktgetrieben, mit unterschiedlich hohen Anteilen der verschiedenen Technologieoptionen oder Eigentümerstrukturen usw.) zu forcieren und in einer Weise zu organisieren, die mit einem pluralistischen Verständnis von Demokratie kompatibel ist. Agonistische Arenen im Handlungsfeld Energiewende könnten im besten Fall die Auseinandersetzung mit ambitionierten sozial-ökologischen Transformationsbestrebungen befördern und gleichzeitig zu einer Ausweitung und Intensivierung demokratischer Aushandlungsprozesse (Flügel-Martinsen und Friedrichs 2019, S. 705 ff.) beitragen sowie der Politikverdrossenheit und der Abkehr von pluralistisch-demokratischen Institutionen entgegenwirken (Mouffe 2000, S. 16 f.).

Auch wenn es aus radikaldemokratischer Perspektive keinen theoretisch begründeten Vorrang einer konkreten Form der Entscheidungsfindung geben kann (Nonhoff 2013, S. 328), werden durchaus Vorschläge für die Bearbeitung von Gemeinwohlkonflikten formuliert. Diese reichen von Skizzen eines linken Populismus, mit dem Parteien links der Mitte für Mehrheiten und Regierungsmacht kämpfen sollen (Mouffe 2018), über die Diskussion des Potenzials von Volksbegehren zur Rekommunalisierung von Stadtwerken und Energienetzen, um eine Demokratisierung der Energiewende voranzutreiben (Haas und Sander 2018), bis hin zur Anregung, Beteiligungsverfahren radikaldemokratisch auszuweiten, so dass auf regionaler Ebene verhandelt werden könnte, in welchem Handlungsfeld (Landwirtschaft, Mobilität, Strom oder Wärme) und mit welchem Strategiemix (Suffizienz, Effizienz oder Zubau von erneuerbaren Energien) verbindliche Emissionsreduktionen erreicht werden sollen (Barry und Ellis 2011). Neben diesen Vorschlägen zur Ausschöpfung institutionalisierter Möglichkeiten der Einflussnahme gibt es in radikaldemokratischen Arbeiten eine Affinität zu Praktiken des zivilen Ungehorsams, die als konstituierende Macht verstanden werden, d. h. als Mittel zur (Re‑)Politisierung naturalisierter Strukturen (Celikates 2019). Das transformative Potenzial zeigt sich in den Protesten von *Fridays for Future, Ende Gelände* und *Extinction Rebellion*, die zu einer (Re‑)Politisierung der Energie- und Klimapolitik beigetragen haben. In den klimapolitisch motivierten Aktionen zivilen Ungehorsams findet die tendenziell marginalisierte Position (die auch in der Wissenschaft, beispielsweise in Arbeiten zu *Degrowth* und *Politischer Ökologie *vertreten wird), dass es angesichts der Klimakrise einer umfassenden Transformation der politischen und wirtschaftlichen Rahmenbedingungen bedarf, ihren politischen Ausdruck.

## Fazit

Zusammenfassend lässt sich konstatieren, dass auf der Ebene von Regierungspolitik die Trias aus Versorgungssicherheit, Bezahlbarkeit sowie Klima- und Umweltschutz als vermeintlich gleichberechtigte Ziele der Energiewende artikuliert werden. Eine explizite Priorisierung von Zielen bleibt aus, weshalb es in den Konflikten um konkrete Politikinstrumente und Energieinfrastrukturprojekte zu de facto Ziel-Priorisierungen kommt. Dabei hat eine Zweck-Mittel-Umkehrung stattgefunden, so dass der Ausbau der erneuerbaren Energien und der Elektromobilität nicht mehr nur Mittel ist, sondern zum eigentlichen Ziel wird (und beispielsweise Klimaschutzziele nachgeordnet werden). Was durch diese Engführung auf Fragen über Technologie (‑förderung und -akzeptanz) auf der Strecke bleibt, ist eine Verhandlung über Zielkonflikte und miteinander konkurrierende Energiewende-Visionen.

Daraus ergeben sich Probleme, sowohl was das Gelingen der Energiewende selbst als auch was die Effekte auf die politische Kultur betrifft. Erstens bergen vage gehaltene Ziele stets die Gefahr, zu unwirksamen, kontraproduktiven oder teuren Maßnahmen zu führen, wie am Beispiel des Kohleausstiegs erläutert. Zweitens gibt es verstärkt Konflikte auf der Ebene der Umsetzung, weil in den Auseinandersetzungen um die Umsetzung der Energiewende die Konflikte um ihre Ziele ausgetragen werden. Damit zusammenhängend kommt es, drittens, zu Zielverfehlungen, beispielsweise in Bezug auf die Senkung von Treibhausgasemissionen. Viertens artikuliert sich in den Konflikten um die Umsetzung der Energiewende nicht nur die Kritik an einzelnen Entscheidungen, sondern ein allgemeines Misstrauen gegenüber Entscheidungsträger*innen, staatlichen Institutionen und etablierten Entscheidungsfindungsprozessen. In den Energiekonflikten zeigen sich Phänomene einer Demokratiekrise, auf die in den Verhandlungen um konkrete Energieprojekte nicht adäquat reagiert werden kann.

Die Planung und Durchführung von Energieprojekten orientiert sich am Paradigma der deliberativen Demokratie. Allerdings erfüllen die angewendeten Beteiligungsverfahren die formulierten hohen Ansprüche in der Regel nur ungenügend. Darüber hinaus haben deliberative Verfahren notwendigerweise – und nicht nur bei mangelhafter Umsetzung – exkludierende Effekte. Die Orientierung am kontrafaktischen Ideal der Machtfreiheit wirkt sich in der Regel zum Vorteil privilegierter und zum Nachteil von marginalisierten Gruppen aus. Weiterhin können Positionen, die auf eine weitreichende Neuausrichtung des Gemeinwohls zielen, nicht in deliberative Entscheidungsfindungsprozesse eingebracht werden. Aufgrund dieser exkludierenden Effekte und Einschränkungen ergibt sich eine Diskrepanz zwischen den Erwartungen an Deliberationen als machtfreie und inklusive Prozesse und den tatsächlich erfahrenen Beteiligungsverfahren.

Vor diesem Hintergrund lautet meine These, dass die Kombination aus fehlender Bereitschaft (von Akteur*innen der Regierungspolitik) zur Austragung von Konflikten um die Priorisierung von Gemeinwohlzielen der Energiewende plus einseitiger Orientierung am deliberativen Demokratiemodell (bei der Umsetzung von Energieprojekten) die Tendenzen einer Demokratiekrise weiter verschärfen. Das radikaldemokratische Argument, dass die Vermeidung von politischer Konfrontation entweder zu Apathie und Politikverdrossenheit oder, schlimmer noch, zu anti-demokratischen, rassistischen Formen kollektiver Identifizierung führt, zeigt sich im Handlungsfeld der Energiewende in den Phänomen der Legitimations- und Repräsentationskrise sowie der Verknüpfung der Kritik an der (Umsetzung der) Energiewende mit rechtsautoritären bis rechtsextremen Positionen, die pluralistisch-demokratische Institutionen ablehnen. Aus radikaldemokratischer Perspektive besteht die adäquate Antwort auf diese Tendenzen einer Demokratiekrise in der Austragung von Konflikten um die Definition und Priorisierung von Gemeinwohlzielen der Energiewende. Auf diese Weise könnten politische Leidenschaften mobilisiert und kanalisiert werden. In diesem Sinne sollte die Konfrontation zwischen konkurrierenden Energiewende-Visionen forciert und in einer Weise organisiert werden, die mit einem pluralistischen Verständnis von Demokratie kompatibel ist.

## Footnotes

1 Teile der Argumentation dieses Aufsatzes wurden – in einer früheren Fassung – bereits in einem Discussion Paper veröffentlicht (Krüger 2020).

2 Strategien der Suffizienz zielen auf einen absolut geringeren Verbrauch von Energie, Rohstoffen und Land durch eine verringerte Produktion und Konsumtion von Gütern und Dienstleistungen ab.

3 Der Begriff „Senke“ bezieht sich auf ein Ökosystem, das in der Lage ist, Emissionen zu absorbieren. Die wichtigsten Kohlenstoffsenken sind Wälder und Ozeane.

4 Der Begriff geht auf Polanyi (1944) zurück und beschreibt Prozesse der Deregulierung von Märkten, d. h. der tendenziellen Herauslösung von Wirtschaftshandeln aus der staatlichen bzw. gesellschaftlichen Einhegung. Strategien des *disembedding* orientieren sich an der Vorstellung von sich selbst regulierenden Märkten. Umgekehrt zielen Strategien des *embedding *darauf, dass sich wirtschaftliches Handeln stärker an tradierten oder demokratisch ausgehandelten Normen (z. B. Reziprozität) und Werten (z. B. Reduktion von Armut und Ungleichheit) orientiert.

5 Allerdings werden die negativen Aspekte der erneuerbaren Energien in der Regel nur partiell wahrgenommen, die Problematik der Rohstoffimporte und deren zerstörerischen Effekte im globalen Süden werden z. B. kaum thematisiert.
